# A Primary Squamous Cell Carcinoma of the Thyroid Presenting as the Anaplastic Thyroid Carcinoma: A Case Report

**DOI:** 10.3389/fsurg.2020.590956

**Published:** 2020-10-19

**Authors:** Rui-zhe Zheng, Guo-hui Huang, Ying-jie Xu

**Affiliations:** ^1^Department of General Surgery, Shanghai Tongren Hospital, Shanghai Jiao Tong University School of Medicine, Shanghai, China; ^2^Department of Otolaryngology-Head and Neck Surgery, Shanghai Ninth People's Hospital, Shanghai Jiaotong University School of Medicine, Shanghai, China; ^3^Department of Neurosurgery, Shanghai Tenth People's Hospital, Tongji University School of Medicine, Shanghai, China

**Keywords:** primary squamous cell carcinoma of the thyroid, anaplastic thyroid carcinoma, differential diagnosis, early identification, treatment

## Abstract

**Background:** Primary squamous cell carcinoma of the thyroid (PSCCT) is an uncommon malignancy that is difficult to diagnose and differentiate. There is no consensus for the early clinical, radiological, or ultrasonic identification of PSCCT before pathological changes are observed in patients. There is also no suitable treatment due to the absence of a definite diagnosis.

**Case Presentation:** A 76-year-old female patient complained about a rapidly growing cervical mass, dyspnea, dysphagia, and a change in her voice. Based on the results of thyroid ultrasound, fine-needle aspiration, and plain and enhanced CT, the patient was initially diagnosed with anaplastic thyroid carcinoma (ATC). Thereafter, we removed the mass that was the patient's main complaint. The gross examination of the patient's symptoms also supported our previous diagnosis. However, her disease was finally diagnosed as PSCCT, according to the histopathology and immunohistochemistry findings of the mass.

**Conclusion:** Our case highlights the need for a comprehensive framework in the management of PSCCT. The more auxiliary examinations (e.g., ultrasonographic, radiology, or biopsy examinations) we take, the more likely we are to identify this disease. Immunohistochemistry is currently the preferred examination for the diagnosis of PSCCT, while surgical resection combined with radio-sensitizing therapy and adjuvant chemotherapy is the main treatment method for PSCCT.

## Introduction

Both primary squamous cell carcinoma of the thyroid (PSCCT) and anaplastic thyroid carcinoma (ATC) have a lower incidence rate in patients (1, 2); previous reports show that PSCCT has an incidence of <1%, while ATC accounts for 1–2% of all thyroid carcinoma cases (3). Both diseases are more common in the elderly population, with the average age of onset being between 60- and 70-years. PSCCT of the thyroid is also more common in older-aged women between 60- and 70-years old (4). Patients with this disease are usually admitted to the hospital following complaints about a rapidly growing cervical mass, dyspnea, or dysphagia, and changes in their voice. The natural course of these two thyroid carcinomatous pathological types are highly malignant and aggressive and frequently invade the surrounding and distant organs, while the prognosis for these patients is poor (1, 3). PSCCT is an exceedingly aggressive disease with a poor prognosis, and its symptoms are similar to those of ATC (3, 5). The most recurrent symptoms of PSCCT show the same pathogenesis as ATC, namely, hoarseness caused by the recurrent laryngeal nerve involvement, and dysphagia and dyspnea related to mechanical compression (2). Overall, there were approximately 79% of PSCCT cases that were misidentified as ATC in a previous study (3). Due to its high malignancy, regardless of whether the cases are PSCCT or ATC, more than 50% of patients presented regional lymph node metastases, while some of them (25%) presented distant metastases (lung, bone, liver, heart, and kidney) in the early stages of the disease (1). Similar to ATC, the survival rate of patients with PSCCT is <1-year after the diagnosis is given. This may be associated with the size of the tumor (6), the T or N stage, except the M stage, and the squamous carcinoma morphological subtypes. In previous studies, PSCCT was more frequently observed in patients with a higher T stage (the T1 stage accounted for only 5%, while the T4 stage was 70%) and lower the N stage, indicating a reduced survival rate (1). The patients' survival rates were higher for large cell, non-keratinized squamous cell carcinoma and were lower for spindle cell and micro-invasive squamous cell carcinomas. Due to the rarity of the disease, there are only a few cases of PSCCT that are confirmed by the combination of clinical features, thyroid ultrasound, and iconography in the preoperative diagnosis (7). Since the diagnosis of PSCCT requires the diagnosis of exclusion, previous studies aimed at the exclusion of metastases from other primary sites. To date, clinicians continue to experience challenges in the early distinction between PSCCT and ATC.

In this study, we reported the case of PSCCT in a 76-year-old woman who was initially misdiagnosed with ATC, emphasizing the differential diagnosis of such diseases, and we aimed to recommend an optimal treatment for PSCCT.

## Case Presentation

### The Patient's Demographics, Symptoms, Signs, and Medical History

A 76-year-old female patient was admitted to our hospital as the result of progressive enlargement of a left cervical mass that the patient had over the past 2 months, accompanied by pain, dysphagia, and hoarseness. The patient initially found a “mung”-sized mass in her anterior region of the left neck, and her health was in good condition before the discovery of the mass. She had no history of smoking and alcohol consumption.

### Physical Examination

During the physical examination, we found that her trachea deviated to the right side. A fixed and hard neck mass was also found in her left lobar thyroid. The mass had a smooth boundary and could be palpated without sensitivity. The size of her protuberance was ~2 × 2 cm. The mass could also move up and down when she swallowed and was accompanied by disorders of her vocal cord. There was no palpable cervical lymphadenopathy, and the examination results of her nervous system, heart, chest, and abdomen were normal.

### Diagnostic Assessment

**Figure 1:**
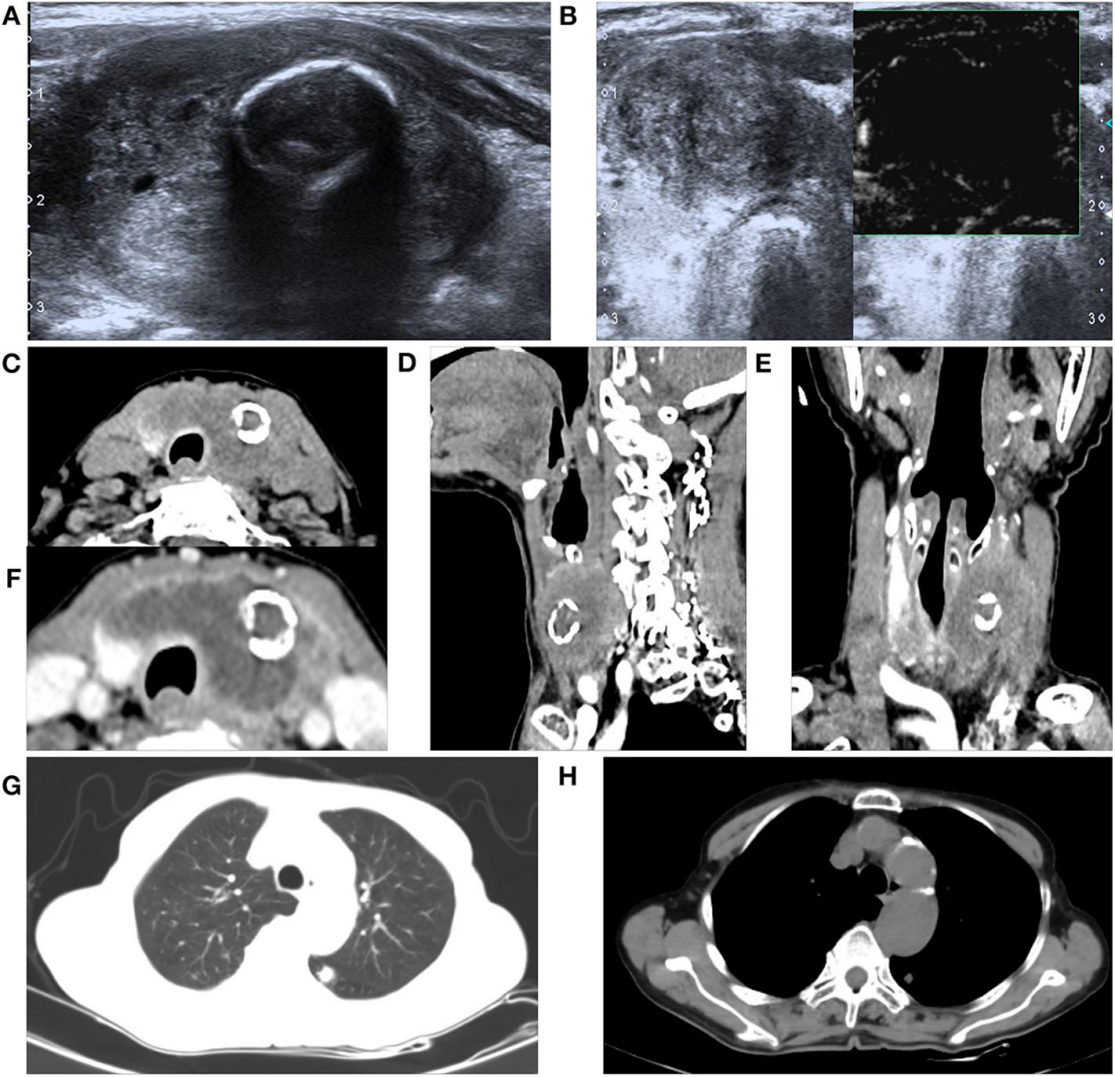
A 76-year-old female with primary squamous cell carcinoma of the thyroid (PSCCT) in the left neck. **(A)** Low signal of grayscale ultrasound shows heterogeneously hypoechoic solid mass sized about 5 × 4 × 3 cm in her left thyroid gland accompanied with a 1.5 × 1.2-cm-size, well-defined, round-like microcalcification located in the central portion. **(B)** Preoperative fine-needle aspiration cytology of the mass shows no definite signs of malignancy. **(C–E)** CT scans show an annular calcification accompanied with a peripheral cystic degeneration portion in her left thyroid gland accompanied with the right deviation of trachea (axial, sagittal, and coronal planes). **(F)** Contrast-enhanced CT shows obvious heterogeneous enhancement with the central non-enhancing necrotic portion, and the thyroid capsule is intact. Chest CT suggested small nodules in the lower lobe of left lung **(G)** pulmonary window and **(H)** mediastinal window.

The thyroid ultrasound showed a well-defined, lobulated, heterogeneous hypoechoic of her left lobe, with a circular hyper-echogenicity measuring of ~1.5 × 1.2 cm. The thyroid capsule appeared to be intact and was labeled in TIRADS (Thyroid Imaging Reporting and Data System) as 3–4a (8). The ultrasound also showed the absence of enlarged lymph nodes in her neck (Figure 1A). Fine-needle aspiration (FNA) cytology showed no definite signs of malignancy (mention Bethesda category) (Figure 1B), and an X-ray showed that the patient's cervical trachea shifted toward the right side. CT showed a hypodense mass occupying her left thyroid gland and across the isthmus to the whole thyroid lobe, accompanied by a ring calcification feature on her left lobe (Figures 1C–E). Contrast-enhanced CT showed a hypodense shadow with an annular calcification in her left lobar thyroid, and the capsule appeared to be intact (Figure 1F). There was also no indication of cervical lymph node metastasis.

**Figure 2:**
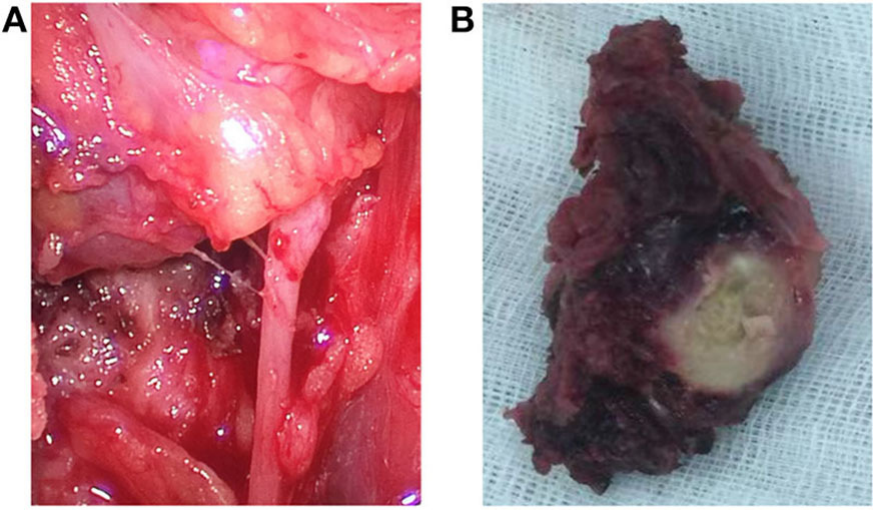
Intraoperative findings and the gross specimens. **(A)** Endoscopic thyroidectomy shows the trachea deviated to the right and the recurrent laryngeal nerve was involved. **(B)** The gross specimen of the left thyroid mass is well-defined, with an approximate size of 5 × 3 × 2.5 cm and a 3 × 2 × 2 cm yellowish necrotic portion accompanied with calcification in the central portion.

The patient's leukocyte count, serum T4, thyroid-stimulating hormone (TSH), thyroglobulin, and serum calcium were all within the normal range, and we were not able to detect the difference in the tumor markers in her blood serum except for elevation of CA199 by three times. Based on the abovementioned results, the patient's mass was considered as a rapidly growing single mass that was accompanied by the nerve recurrent laryngeal nerve invasion and internal necrosis and calcification. Thus, the mass was diagnosed as a poorly differentiated thyroid carcinoma, and we chose to remove it as a result of the abovementioned symptoms. During the course of the operation, there was a 5 × 3 × 2.5 cm mass with extensive adhesion of the topical surface, esophagus, and anterior cervical muscle in her left lobar thyroid (Figure 2A). We also found that the trachea was deviated to the right and that there was recurrent laryngeal nerve involvement. Total thyroidectomy was performed, and the central lymph node dissection was additionally performed since the intraoperative frozen biopsy suggested malignancy. No adjacent damage to the organs, such as the esophagus and trachea, were detected during the operation. The gross specimen showed a well-defined mass in the left thyroid gland, which consisted of large and extensive necrosis with an internal gray-yellow and hard texture nodule (Figure 2B). There was also a cystic lesion accompanied by partial calcification within the mass.

**Figure 3:**
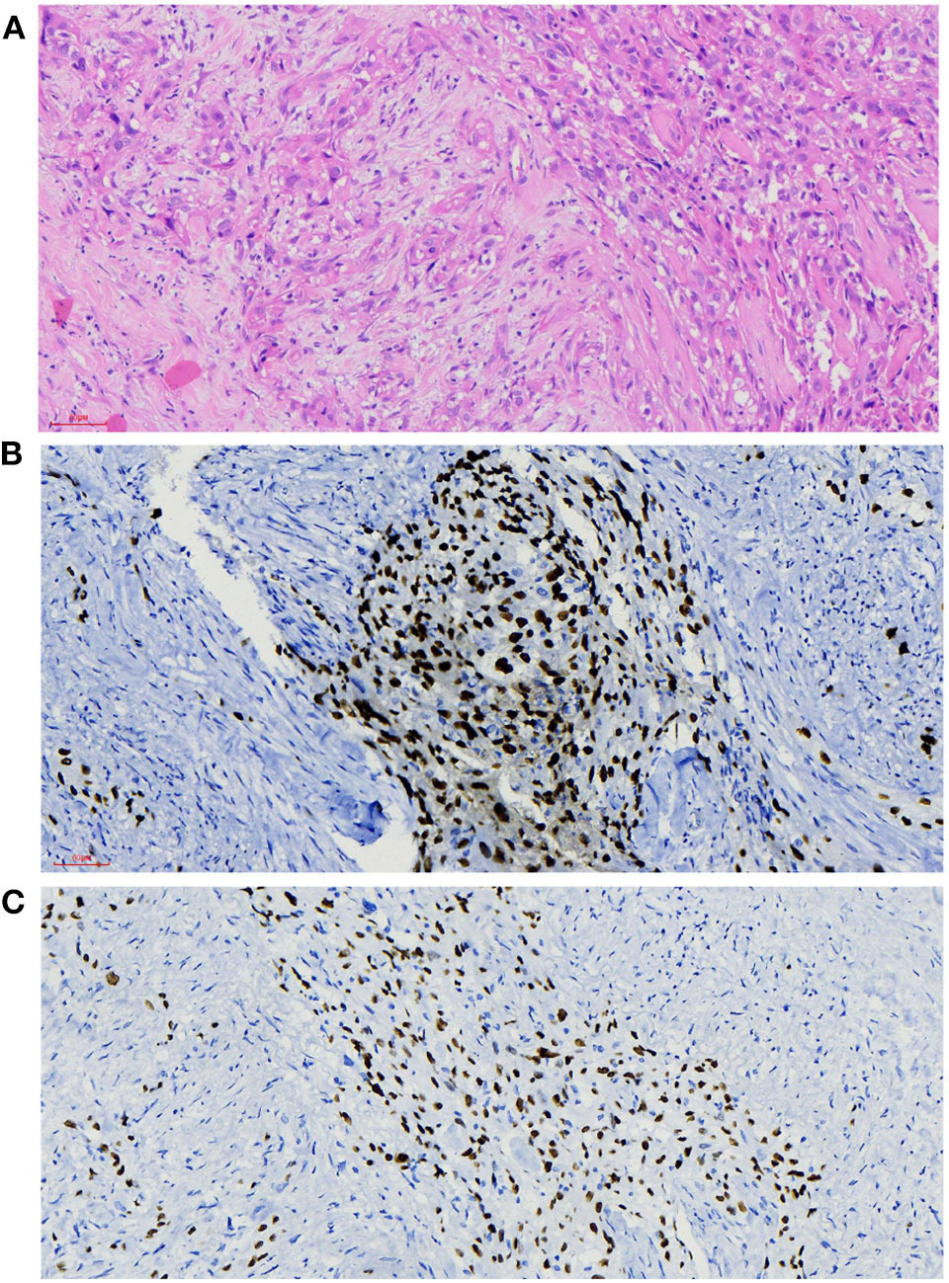
Histopathological findings. **(A)** Microscopy observed that nesting pattern with cornified pearl, keratin, and intercellular bridge (H&E, ×20). **(B,C)** Immunochemistry shows primary squamous cell carcinoma of the thyroid (PSCCT) cells positive for p63 and p40 (p63 and p40 immunostaining, ×20).

The histopathological manifestations of the carcinomas were a prominent nesting pattern, with intercellular bridges and keratinization with a cancer pearl (Figure 3A). This solid epithelial neoplasm also had hyper-chromatic nuclei and prominent eosinophilic nucleoli. During the immunohistochemistry staining of the thyroid mass (Figures 3B,C), her positive stains were keratin (100%), especially AE1/AE3 and CK7, Ki-67 (30%), GAL-3, P63, and P40, while her negative stains were CK20 and calcitonin, CD56 and CD5, thyroglobulin, and thyroid peroxidase. To obtain a better understanding of the exclusion of metastases from other primary sites, we performed lung CT, laryngoscopy, and tracheoscopy. After these examinations were completed, PSCCT was found in her thyroid gland only. We finally interpreted these histopathological characteristics as highly differentiated PSCCT according to the results of the histopathology and immunohistochemistry examinations. Preoperative and postoperative laryngoscopy indicated that the mucous membrane of bilateral vocal cords was smooth, with normal activity and no neoplasm.

### Follow-Up

During the follow-up, the patient's swallowing and phonation were stable, and she was discharged from the hospital on the third postoperative day. After 3 months, she revisited our hospital with a complaint about a pulmonary disease with dyspnea, chest discomfort, and coughing. The chest CT scan indicated multiple, unclear contour and clouding margin lung nodules, about 6–8 mm in size on both sides of her lungs. Along with increased bronchovesicular shadows, we did not observe any enlarged lymph nodes in her mediastinum. However, there was thickening soft tissue around the trachea that was included in the main CT results. After receiving short treatment for respiratory system diseases, the patient died from respiratory failure. CT report before death was as follows: a little inflammation in both lungs, nodules in both lungs, arteriosclerosis of aorta and coronary artery, and a little pericardial effusion, considered as pulmonary metastasis. The patient died due to old age, emaciation, and poor nutritional status.

Intraoperative whole-body CT scan and gastroscopy showed no positive findings, and laryngoscopy showed no positive findings. Chest CT alone suggested small nodules in the lower lobes of both lungs (Figures 1G,H). There were a few fibrous foci in the upper lobe, lower lobe, and tongue segment of the upper lobe of the right lung; aortic and coronary arteriosclerosis; and a small amount of pericardial effusion. No positive findings were found on chest CT and no positive findings on gastroscopy and colonoscopy, and no abnormal descriptions of blood tumor indicators were found. Primary thyroid cancer was considered.

## Discussion

An effective examination for diagnosis must follow the underlying differences in etiologies between PSCCT and ATC. The origin of the primary squamous cell carcinoma became a controversial matter due to the absence of squamous epithelium in the normal gland (1, 8). At present, three hypotheses mostly account for the etiology of PSCCT. The first hypothesis is the “embryonic rest theory,” which assumes that embryonic remnants containing squamous cells (e.g., thymic epithelium, ultimobranchial body, or thyroglossal duct) evolved into squamous cells during embryonic development. According to the second hypothesis, the “metaplasia theory” holds the view that squamous metaplasia originated from potential pathology (e.g., chronic inflammation or Hashimoto thyroiditis), whereas the third hypothesis considers that the squamous differentiated into other histopathological types such as papillary, follicular, or medullary carcinoma (1, 9). At this point, these etiological hypotheses differ from those of ATC. It is also assumed that a normal follicular cell directly transforms into an undifferentiated cell, as opposed to the differentiation into normal cells in ATC patients. Also, ATC always originated in an abnormal thyroid gland and may coexist with differentiated thyroid cancer, which can be classified into papillary or follicular thyroid carcinoma (2). In previous studies, more than 80% of ATC patients have a long history of goiter while PSCCT patients have rapidly enlarging masses (8).

Imaging using ultrasonography (US) to obtain a definite diagnosis of PSCCT has not been ruled out. However, in terms of previous US imaging findings, PSCCT was described as the eggshell calcification nodule, surrounded by soft tissues or the hypoechoic solid nodules with irregular margins (8, 10), showing a risk of malignancy in ultrasonic diagnostic examinations (10, 11). In our case, the hypoechoic mass with diffuse microcalcifications revealed the malignancy of the pathological features. We also conducted FNA cytology on our patient to confirm the pathological type of the thyroid nodule. The absence of a malignant base in the FNA examination may have been due to the desmoplastic and fibrotic reaction that firmly held the carcinoma cells. Consequently, the diseased cells become harder to obtain (12). Even if those carcinoma cells have been picked up by FNA, they would be washed away while making the pathological sections of specimen (8). Another possible reason may be that we had a puncture to the normal follicular tissue or infiltration in the location of the lymphocyte. To our knowledge, the most aggressive forms of thyroid cancer are solid and have a marked hypoechogenicity and an irregular margin that is accompanied by internal calcification (13). It was reported that any type of calcification detected by US shows twice the risk of malignancy. Thus, we started with the diagnosis of a highly malignant tumor in our case.

Considering the limitations of US in preoperative evaluations, CT was performed. In our CT findings, PSCCT was described as a huge mass containing focal cystic changes and the course/curvilinear or eggshell calcification, which can change the pressure of the trachea (8, 14). The imagine features of eggshell or peripheral calcification was defined as a poorly curvilinear echogenic structure that was encompassed by 120° or more for the circumferences (15). In our case, an annular calcification might indirectly reflect the malignant degree of a carcinoma. Radiologically, there was advanced infiltration and effacement of the neighboring structures in our case. According to the invasion of the esophagus, the displacement of the trachea, and the enclosure of the adjacent vessels, we assumed that our case was a presentation of ATC. In the previous studies, there are ~97% of ATC patients with a rapidly enlarging thyroid mass that is accompanied by a compressed and dislocated trachea (2). Approximately 70% of patients with ATC also have a metastasis of the adjacent organs (including the muscles, trachea, esophagus, and larynx) during CT examinations. At this point, PSCCT is not completely different from ATC in its invasiveness. According to previous reports, radiological investigations cannot exactly identify PSCCT (5). However, to eliminate the possibility of secondary metastasis, it is necessary to use radiologic technology to detect the other organs in the patient's neck, such as the pharynx, trachea, and esophagus.

No other obvious abnormality was found in the patient's blood, and the biochemical detection can suggest the characteristics of the tumors. Although there are many established biomarkers that are involved in malignant tumors in the head and neck (16), thyroglobulin (TG) is currently the only indicator of thyroid cancer (17). It has been reported that the principal immunohistochemical markers of serum TG always have a negative response in patients with ATC, while they also have a weak or ambiguous positive response in PSCCT (2, 18). Unfortunately, the findings derived from serum biomarkers (thyro-peroxidase, TSH receptor, and sodium/iodide symporter) can barely be used to clarify the diagnosis of PSCCT.

In PSCCT, a pathological examination is often used to confirm its diagnosis. The microscopic description of PSCCT is composed of squamous cells, with prominent intercellular bridges in the different differentiation states (12). In PSCCT patients, p53 and Ki-67 are established as poor prognostic indicators, while playing an important role in neoplastic proliferation, as well as the squamous metaplastic process (14). The p53 and Ki-67 staining of 30% showed poor prognostic features in our case. CK5/6 was used to define poorly differentiated metastatic carcinomas and acted as a predictor of a primary tumor with a squamous epithelial origin (14, 19). CK7 and AE1/AE3 positive staining supported the thyroid follicular epithelial rather than pharyngeal or laryngeal origin. Similar to the results found in previous studies, the positive expression of TTF-1 nuclear staining provides proliferative and neoplastic evidence for thyroid tissues, while the immunity reaction is rare in ATC patients (14, 20). The positivity of CK7 and TTF-1 means that the focus originated from the thyroid gland, which also provides evidence of the thyroid follicular epithelial, rather than the origin of an extra thyroidal source (laryngeal or oropharyngeal) or branchial pouch remnants. Also, negative CD5 stains mean differential diagnosis of rare intrathyroidal neoplasms, similar to carcinomas showing thymus-like differentiation (14). The transcription factor, PAX8, is also positively reported in most PSCCT patients (21, 22). Although the positive expression of PAX8 means that there is a developmental correlation between PSCCT and ATC (21), by comparison, the expression for PSCCT is almost 100% of cases while the expression of ACT is 50% of cases. In recent years, some additional immunohistochemistry markers for the differential diagnosis of these diseases have included a pan-keratin cocktail, calcitonin, and GATA3 (staining in 50% of cases) (23). If they were validated in further studies, these markers may be beneficial in the differential diagnosis of these diseases. Therefore, the biomarkers remain the most helpful markers for the histopathological evaluation and are by far the most reliable diagnostic method for PSCCT in patients (2, 24).

Currently, there is no standard treatment that can be used as a guideline for PSCCT due to the rarity of the cases (5, 6). The current treatment modality also resembles that of ATC (1). The indications of treatment for PSCCT primarily include pain and functional impairment management or the management of the complications that occur in patients before a definite diagnosis is achieved. Although the prognosis of PSCCT is unfavorable, regardless of the treatment, complete resection is significant in improving the patients' survival. In previous studies, the 3-year survival rate in patients after complete resection is 43.1%, whereas the rate for incomplete resection is 15.9% (5). To our knowledge, the scope of PSCCT's surgical resection is unclear, and the extensive invasiveness of squamous cell carcinomas may be a key factor in surgical failure. Generally, accurately prolonging the excision extension and the extended lymph node dissection is the primary choice for surgeons.

Given that PSCCT is insensitive to chemotherapy and is relatively resistant to radiotherapy, whether to use additional adjuvant therapy or chemotherapy during the perioperative period is controversial (1, 5, 6). To date, surgical resection combined with adjuvant postoperative radiotherapy appears to be the most optimal therapeutic option (1, 5, 7, 25). In future analyses, more details such as local or distant metastasis that mainly affect the disease prognosis should be examined.

## Conclusion

Differentiating the difference between PSCCT and ATC in the preoperative diagnostic period is challenging since both diseases present similarities in the clinical symptoms (Table 1). So far, positive immunohistochemical staining for specific thyroid markers (e.g., PAX8, TTF-1, and p53) may be the only critical method for the differential diagnosis of PSCCT and ATC. Even so, surgical resection should be performed before the definite diagnosis is given, as it takes time to complete the histology, immunohistochemistry, and electron microscopy that are required to conclusively identify PSCCT. A comprehensive perioperative therapeutic strategy including comprehensive surgery, adjuvant chemotherapy, and radiotherapy is crucial in improving the prognosis of PSCCT patients. However, the formation of an appropriate treatment method relies on early identification.

**Table 1:** Differences between PSCCT and ATC.

	**PSCCT**	**ATC**
Etiology	(1) “Embryonic rest theory” (2) “Metaplasia theory” (3) Squamous differentiation	Direct transformation of a normal follicular cell to a completely undifferentiated cell
Clinical manifestation	A rapidly enlarging neck mass observed in older patients (60%), followed by symptoms of dyspnea, dysphagia, or hoarseness (20%) and change of voice (15%).	A rapidly enlarging thyroid mass ranging from 3 to 20 cm in size; the most frequent symptoms are hoarseness, dysphagia, dyspnea, stridor, and cervical pain
Features	(1) Rapidly enlarging mass (2) The mass may associate with other thyroid malignancies (3) Histological features of intercellular bridges and keratin	(1) Rapidly enlarging mass with the volume doubling within 1 week (2) Systemic symptoms (anorexia, weight loss, and shortness of breath) (3) Distant metastases: lung (most common), bone and brain
US findings	Eggshell calcification and peripheral soft tissue or slowly growing, irregularly marinated hypoechoic solid nodule	Solid, marked hypoechogenicity, irregular margin and internal calcification
CT findings	A huge mass containing focal cystic changes, the course/curvilinear or eggshell calcification	A huge mass containing calcification and necrosis and had heterogeneous attenuation, accompanied with the metastasis of adjacent organs
IHC staining	Positive: TTF-1, PAX8, TG, p53, Ki-67 (30%), AE1/AE3, CK5/6, CK7 Negative: CK20, CD5, calcitonin, SYN	Positive: p53, Ki-67, CK8, CK18, CK199, PAX8 (0–50%), GATA3 (50%), low-molecular-mass CK, calcitonin Negative: TG, TTF-1

*PSCCT, primary squamous cell carcinoma of the thyroid; ATC, anaplastic thyroid carcinoma; US, ultrasonographic; CT, computed tomography; IHC, immunohistochemistry*.

## Data Availability Statement

All datasets presented in this study are included in the article/supplementary material.

## Ethics Statement

Written informed consent was obtained from the individual(s) for the publication of any potentially identifiable images or data included in this article.

## Author Contributions

R-zZ conceived the idea and conceptualized the study. G-hH collected and analyzed the data. R-zZ and Y-jX drafted the manuscript. G-hH reviewed the manuscript. All authors read and approved the final draft.

## Conflict of Interest

The authors declare that the research was conducted in the absence of any commercial or financial relationships that could be construed as a potential conflict of interest.
